# Supplementary material for: Identification of Freezing-Responsive microRNAs and Their Targets in Chinese Jujube by Small RNA and Degradome Sequencing
Source: Int J Mol Sci. 2026 Sep 4;27(17):7912. doi: 10.3390/ijms27177912 (PMC13566890; doi:10.3390/ijms27177912)
Supplement: Supplementary file 1 [file ijms-27-07912-s001.zip › Supplementary Material S2/novel_miR_142.pdf]

Provisional ID	:	novel_miR_142
Score total	:	900.9
Score for star read(s)	:	3.9
Score for read counts	:	894.4
Score for mfe	:	1
Score for randfold	:	1.6
Score for cons. seed	:	
Total read count	:	1766
Mature read count	:	1748
Loop read count	:	0
Star read count	:	18

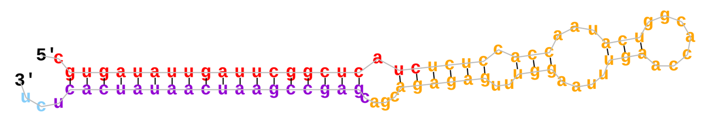

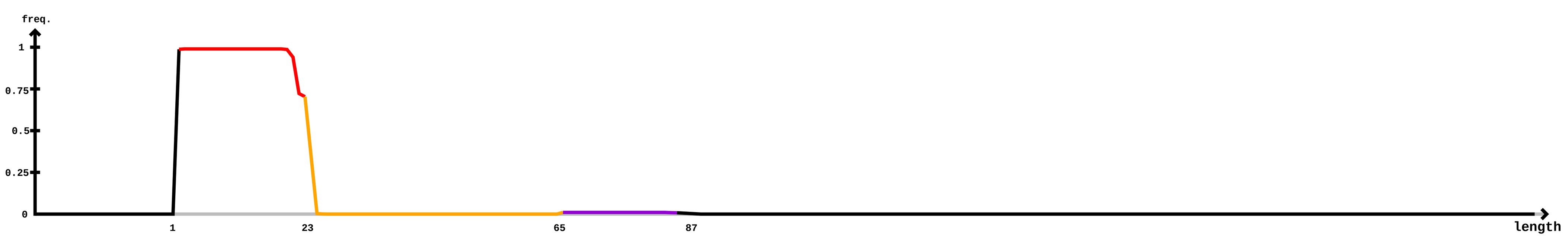

Mature **Star**

```
5'- cauucugcagaaguagacauggcgugauauugauucggcucaucucuccaccaauacuggcaccaaguuuaagguuugagagacgacgagccgaaucaauaucacucuuguaugcuucuucgcauauuaucaagguuuuuccuguuuuuugaguuccaauauuagccaagaagcaucucugcaggcaacucaugcauuaagcuacauagaagcuuguuuuggcucuccaucccccucuuguaaagucuuc -3'   obs
    cauucugcagaaguagacauggcgugauauugauucggcucaucucuccaccaauacuggcaccaaguuuaagguuugagagacgacgagccgaaucaauaucacucuuguaugcuucuucgcauauuaucaagguuuuuccuguuuuuugaguuccaauauuagccaagaagcaucucugcaggcaacucaugcauuaagcuacauagaagcuuguuuuggcucuccaucccccucuuguaaagucuuc        exp
    ....((((((((.........((.((((((((((((((((((.((((((.(((...(((.......)))....)))..))))))....)))))))))))))))))).)).((((((......))))))......((.....))((((((((((.(((........))))))))))))..))))))))(((.....)))..((((((.......))))))....((((...((.........))...))))...
```

read	reads	mm	sample
cgugauauugauucggcu	1	0	LT-1
cgugauauugauucggcu	5	0	LT-3
cgugauauugauucggcuU	1	1	LT-3
cgugauauugauucggcuc	7	0	LT-1
cgugCuauugauucggcuc	1	1	LCK-3
cgugauauugauucggcuc	33	0	LCK-3
cgugauauugauucggcuc	21	0	LT-2
cgugauauugauucggcuA	6	1	LCK-3
cgugauauugauucggcuc	13	0	LT-3
cgugauauugauucggcuca	9	0	JCK-2
cgugauCuugauucggcuca	1	1	LT-3
cAugauauugauucggcuca	1	1	LT-2
cgugauauugauucggcuca	71	0	LT-2
cgugauauugauucggcuca	103	0	LCK-3
cgugauauugauucggcuca	3	0	JCK-3
cgugauauugauucggcuca	2	0	JCK-1
cgugauauugauucggcuca	48	0	LCK-1
cgugauauugauucggcucU	3	1	LT-2
cgugauauugauucggcuca	23	0	LT-1
cgugauauugauucggcuca	52	0	LT-3
cgugauCuugauucggcuca	1	1	LCK-1
cgugauauugauucgAcuca	1	1	LCK-3
cgugauauugauucggcuca	1	0	JT-2
cgugauauugauucggcuca	3	0	JT-3
cgugauCuugauucggcuca	1	1	LCK-2
cgugauauugauucggcuca	60	0	LCK-2
cgugauCuugauucggcucau	1	1	LT-3
cgugauauugauucggcucaA	1	1	LT-3
cgugauauugauucggcucau	1	0	LT-3
cgugauauugauucggcucau	8	0	LT-1
cgugauauugauucggcucau	14	0	LCK-3
cgugauauugauucggcucau	9	0	LT-2
cgugauauugauucggcucauU	3	1	LT-1

```
                      Mature                                                                Star
cauucugcagaaguagacauggcgugauauugauucggcucaucucuccaccaauacuggcaccaaguuuaagguuugagagacgacgagccgaaucaauaucacucuuguaugcuucuucgcauauuaucaagguuuuuccuguuuuuugaguuccaauauuagccaagaagcaucucugcaggcaacucaugcauuaagcuacauagaagcuuguuuuggcucuccaucccccucuuguaaagucuuc
......................Agugauauugauucggcucauc..............................................................................................................................................................................................................   1     1     LT-1
......................cgugauauugauucggcucauU..............................................................................................................................................................................................................   1     1     JCK-2
......................cguAauauugauucggcucauc..............................................................................................................................................................................................................   1     1     JCK-3
......................cgugauauugauucggcucauU..............................................................................................................................................................................................................   5     1     LT-3
......................cgugauauugauucggcucauU..............................................................................................................................................................................................................   13    1     LT-2
......................cgugauauugauucggcucauU..............................................................................................................................................................................................................   1     1     JCK-1
......................cgugauauugauucggcucauc..............................................................................................................................................................................................................   99    0     JCK-2
......................cgugauauugauucggcucauU..............................................................................................................................................................................................................   5     1     JCK-3
......................cgugCuauugauucggcucauc..............................................................................................................................................................................................................   2     1     JCK-2
......................cgugauauugCuucggcucauc..............................................................................................................................................................................................................   1     1     JCK-1
......................cgugauauugauucggcucauc..............................................................................................................................................................................................................   31    0     LCK-1
......................cgugauauugauucggcucauU..............................................................................................................................................................................................................   9     1     LCK-3
......................cgugauauugauucggcucauc..............................................................................................................................................................................................................   51    0     JCK-1
......................cgugCuauugauucggcucauc..............................................................................................................................................................................................................   1     1     LT-3
......................cgugauauugCuucggcucauc..............................................................................................................................................................................................................   1     1     LT-1
......................cgugauauugauucggcucCuc..............................................................................................................................................................................................................   1     1     LT-2
......................cgugauauugCuucggcucauc..............................................................................................................................................................................................................   1     1     JCK-2
......................cgugauauugauucggcucauc..............................................................................................................................................................................................................   122   0     LT-2
......................cgugauauugauucggcCcauc..............................................................................................................................................................................................................   1     1     LT-1
......................cgugCuauugauucggcucauc..............................................................................................................................................................................................................   1     1     LCK-3
......................cgugauauugauucggcucauc..............................................................................................................................................................................................................   237   0     LT-1
......................cgugauauugauucggcucauc..............................................................................................................................................................................................................   137   0     LCK-3
......................cNugauauugauucggcucauc..............................................................................................................................................................................................................   1     1     JCK-1
......................cgugauauugauucggcucauc..............................................................................................................................................................................................................   93    0     JCK-3
......................cguUauauugauucggcucauc..............................................................................................................................................................................................................   1     1     LT-2
......................cgugauauugauucggcucauc..............................................................................................................................................................................................................   31    0     LCK-2
......................cgugauauugauucggcucauc..............................................................................................................................................................................................................   248   0     LT-3
......................cgGgauauugauucggcucauc..............................................................................................................................................................................................................   1     1     LT-2
......................cgugauauugauucggcucauAu.............................................................................................................................................................................................................   1     1     LT-2
......................cgugauauugauucggcucaucA.............................................................................................................................................................................................................   1     1     LCK-3
......................cgugauauugauucggcucaucu.............................................................................................................................................................................................................   34    0     LT-3
......................cgugauauugauucggcucaucu.............................................................................................................................................................................................................   4     0     JCK-3
......................cgugauauugauucggcucauUu.............................................................................................................................................................................................................   1     1     LT-3
......................cgugauauugauucggcucaucA.............................................................................................................................................................................................................   3     1     LT-2
......................cAugauauugauucggcucaucu.............................................................................................................................................................................................................   1     1     LT-2
......................cgugauauugauucggcucaucu.............................................................................................................................................................................................................   3     0     JCK-2
......................cgugauauugauucggcucaucu.............................................................................................................................................................................................................   15    0     LCK-3
......................cgugauauugauucggcucaucu.............................................................................................................................................................................................................   17    0     LT-2
......................cgugauauugCuucggcucaucu.............................................................................................................................................................................................................   1     1     LT-1
......................cgugauauugauucggcucauUu.............................................................................................................................................................................................................   9     1     LCK-3
......................cgugauauugauucggcucaucu.............................................................................................................................................................................................................   30    0     LT-1
......................cgugauauugauucggcucaucA.............................................................................................................................................................................................................   2     1     LT-1
......................cgugauauugauucggcucaucu.............................................................................................................................................................................................................   1     0     JCK-1
......................cgugauauugauucggcucauUu.............................................................................................................................................................................................................   6     1     LT-2
......................cgugauauugauucggcucaucA.............................................................................................................................................................................................................   3     1     LT-3
......................cgugauauugauucggcucauUu.............................................................................................................................................................................................................   2     1     LT-1
......................cgugauauugauucggcucaucuU............................................................................................................................................................................................................   1     1     JCK-2
......................cgugauauugauucggcucaucuU............................................................................................................................................................................................................   1     1     LT-2
......................cgugauauugauucggcucaucuU............................................................................................................................................................................................................   2     1     LT-3
......................cgugauauugauucggcucaucuUu...........................................................................................................................................................................................................   1     1     LCK-3
.......................gugauauugauucggcuca................................................................................................................................................................................................................   1     0     LT-2
.......................gugauauugauucggcucauc..............................................................................................................................................................................................................   2     0     LT-3
.......................gugauauugauucggcucauc..............................................................................................................................................................................................................   1     0     LT-1
......................................................................................cgagccgaaucaauaucU..................................................................................................................................................   2     1     LCK-3
......................................................................................cgagccgaaucaauauca..................................................................................................................................................   1     0     LT-2
......................................................................................cgagccgaaucaauaucac.................................................................................................................................................   1     0     LT-1
......................................................................................cgagccgaaucaauaucacu................................................................................................................................................   6     0     LT-1
......................................................................................cgagccgaaucaauaucacu................................................................................................................................................   2     0     LT-3
......................................................................................cgagccgaaucaauaucacu................................................................................................................................................   5     0     LT-2
......................................................................................cgagccgaaucaauaucacuU...............................................................................................................................................   1     1     LT-1
```
